# Prognostic factors for mobility in children with osteogenesis imperfecta

**DOI:** 10.1097/MD.0000000000030521

**Published:** 2022-09-09

**Authors:** Kenta Sawamura, Hiroshi Kitoh, Hiroshi Kaneko, Akiko Kitamura, Tadashi Hattori

**Affiliations:** a Department of Orthopedic Surgery, Aichi Children’s Health and Medical Center, Obu, Japan; b Department of Comprehensive Pediatric Medicine, Nagoya University Graduate School of Medicine, Nagoya, Japan.

**Keywords:** Hoffer classification, intramedullary nail fixation, mobility, osteogenesis imperfecta

## Abstract

Osteogenesis imperfecta (OI) is a connective tissue disorder characterized by bone fragility. Although the disease severity is known to influence the ability to walk, little is known about how children with severe OI can achieve practical ambulation (PA). This study aimed to determine the early predictors of future mobility in children with OI. Thirty OI patients with an average age of 12.1 years were classified into the PA group (22 patients) and nonambulator group (NA group: 8 patients) on the basis of the Hoffer classification. Various clinical parameters related to mobility were compared between the PA and NA groups. Therapeutic interventions were also compared between the 2 groups. The mean age at diagnosis and initial fracture were significantly lower in the NA group than in the PA group. The height was significantly smaller in the NA group than in the PA group at all ages examined (at birth, 3 years, and 6 years). The number of patients with respiratory failure was significantly higher in the NA group than in the PA group. The age at initial corrective osteotomy of the lower extremities in the PA group was significantly lower than that in the NA group, although there was no significant difference in the disease severity in infancy between the groups. Height during infancy, age at initial fracture, and neonatal respiratory status could be prognostic factors for mobility in OI. Surgical interventions at an early age may influence walking ability in children with moderate OI.

## 1. Introduction

Osteogenesis imperfecta (OI) is a widely variable genetic disorder characterized by increased bone fragility, long bone deformities, short stature, and various connective tissue dysfunctions. The nature and severity of the disease vary significantly among individuals: some cases of severe disease are lethal during the perinatal period, and other cases with milder disease are diagnosed after adolescence due to accidental fractures.^[1,2]^ Current treatment modalities for OI in children include intermittent administration of bisphosphonate (BP) and corrective osteotomies for long bone deformities; however, definite criteria for surgery or medication have not been proposed, and no consensus exists on the effectiveness of these treatments.

Repeated fractures, ligament laxity, muscle weakness, and lower extremity deformities impair the ability to walk in OI. The mobility of children with OI is correlated with the severity of the disease; however, it is difficult to determine at the time of diagnosis. The prognosis for mobility deficit in children with OI is of clinical interest in setting specific treatment and rehabilitation goals. In this study, we investigated the early prognostic factors that influence mobility in children with OI and examined the effects of orthopedic interventions on patient mobility.

## 2. Methods

This study received approval from the institutional ethics review board of Aichi Children’s Health and Medical Center. The medical records of OI patients who were diagnosed clinically and treated at our institution between 2003 and 2020 were retrospectively reviewed. Patients who were diagnosed at 6 years of age or older, those who were younger than 6 years of age at the time of this survey, and those with insufficient perinatal medical information were excluded. Of the 42 consecutive patients, 4 were excluded since they were diagnosed at more than 6 years of age, 6 were excluded since they were younger than 6 years at the time of the survey, and 2 were excluded due to lack of perinatal information. Thus, 30 patients, including 19 boys and 11 girls with an average age of 2.1 years at diagnosis and 12.1 years at the time of this survey, were investigated.

The clinical variables related to mobility included height (standard deviation [SD]) at birth, 3 years, and 6 years, birth weight, bone mineral density at diagnosis measured by dual-energy X-ray absorptiometry of the lumbar spine, neonatal respiratory insufficiency evaluated during neonatal intensive care unit (NICU) management, presence of fracture at birth, age at initial fracture, and treatment history with BP or surgery. Mobility was assessed according to the Hoffer Functional Ambulation Classification.^[3]^ The patients were classified into 4 groups: community ambulator (CA), if the patient could walk outside for most of their daily activities; household ambulator (HA), if the patient used assistive devices and did not walk outside the house; nonfunctional ambulator (NFA), if the patient could walk only during training; and nonambulator (NA), if the patient always needed a wheelchair for transfer.^[4]^ In addition, CA and HA were together categorized as the practical ambulation (PA) group, whereas NFA and NA were categorized as the NA group. PA means patients who can walk and move in daily living, whether indoors or outdoors. Clinical variables during infancy and early childhood were compared between the PA and NA groups. Therapeutic interventions were also compared between the 2 groups. All data were evaluated using SPSS version 26.0 (IBM Corp., Armonk, NY). The Mann–Whitney *U* test and Fisher’s exact test were used for statistical analyses. Differences were considered statistically significant at *P* < .05.

## 3. Results

Among the 30 patients, 15, 7, 2, and 6 were classified as CA, HA, NFA, and NA, respectively, resulting in 22 cases in the PA group and 8 cases in the NA group. The mean age at diagnosis in the PA group (2.9 years) was significantly higher than that in the NA group (0.3 years) (*P* < .01). The mean age at occurrence of initial fracture in the PA group (2.7 years) was also significantly higher than that in the NA group (0.3 years) (*P* < .01). Neonatal fractures were confirmed in 75% of the participants in the NA group and only 10% of those in the PA group (*P* < .01). The mean SD scores for height in the PA group were −0.6 SD at birth, −1.9 SD at 3 years, and −1.7 SD at 6 years, respectively. The corresponding scores in the NA group were −3.5 SD at birth, −5.0 SD at 3 years, and −5.7 SD at 6 years, respectively. Children in the PA group were significantly taller than those in the NA group in all age groups (*P* < .01). In addition, children in the NA group required significantly more NICU management for respiratory insufficiency than those in the PA group (*P* < .01). However, there were no significant differences in birth weight or bone mineral density between the 2 groups (Table 1).

**Table 1:** Comparison between the practical walking group and the nonambulatory group.

Clinical variables	PA (n = 22)	NA (n = 8)	*P* value
Age (years)			
At diagnosis	2.9 ± 2.0	0.3 ± 0.6	<.01
At initial fracture	2.7 ± 2.7	0.3 ± 0.5	<.01
Height (SD score)			
At birth	−0.6 ± 0.8	−3.5 ± 1.4	<.01
At 3 years	−1.9 ± 1.4	−5.0 ± 2.0	<.01
At 6 years	−1.7 ± 1.4	−5.7 ± 2.5	<.01
Weight at birth (g)	2784 ± 371	2386 ± 378	.07
Neonatal respiratory failure (n [%])	3 (14%)	7 (88%)	<.01
Fracture at birth (n [%])	4 (18%)	6 (75%)	<.01
BMD (Z score)	−3.8 ± 2.0	−5.0 ± 1.8	.17
BP treatment (n [%])	18 (82%)	8 (100%)	.55

BMD = bone mineral density, BP = bisphosphonate, NA = nonambulatory, PA = practical ambulation, SD = standard deviation.

BP treatment was performed in all patients in the NA group and in 82% of those in the PA group. Because the BP was administered to almost all cases in the current study, we were unable to evaluate the effect of medication on mobility of OI children. Corrective osteotomies with intramedullary nail fixation of the long bones of the lower extremities were performed in 5 patients (23%) in the PA group and 6 (75%) in the NA group. Next, we compared the patients who underwent surgeries in the former and latter groups. Birth height tended to be smaller in the NA group, but the difference was not statistically significant. Also, the age at diagnosis did not differ between the PA and NA groups in the patients who underwent surgeries. On the other hand, the mean age at initial surgery in the PA group (2.7 years) was significantly lower than that in the NA group (5.1 years) (*P* = .02). Four of the 5 patients in the PA group underwent initial surgery before they started to walk. In addition, the mean height at the time of initial surgery was significantly smaller in the NA group (−5.8 SD) than in the PA group (−3.5 SD) (*P* =.03) (Table 2).

**Table 2:** Comparison of patients who underwent corrective osteotomy and intramedullary nail fixation of lower extremities.

Clinical variables	PA (n = 5)	NA (n = 6)	*P* value
Age (years)			
At diagnosis	0.2 ± 0.3	0.1 ± 0.1	.25
At initial surgery	2.7 ± 0.8	5.1 ± 2.2	.02
Height (SD score)			
At birth	−2.2 ± 1.9	−3.4 ± 2.2	.09
At initial surgery	−3.5 ± 1.0	−5.8 ± 2.3	.03
Number of bones operated	3.8 ± 0.9	3.3 ± 1.0	.46

NA = nonambulatory, PA = practical ambulation, SD = standard deviation.

We defined −4.0 SD as the cutoff value of height for predicting the prognosis of ambulation. A height cutoff of −4.0 SD at birth accounted for 22 of the 24 cases in the PA group, whereas the same cutoff of −4.0 SD at 3 years of age accounted for 22 of 23 cases in this group. Conversely, all 6 children with a birth height of less than −4.0 SD and all 7 patients with a height of less than −4.0 SD at 3 years of age fell in the NA group.

## 4. Discussion

The mobility of children with OI is generally considered to be correlated with disease severity. Using the Sillence classification,^[5]^ Daly et al^[6]^ and Engelbelt et al^[7]^ reported the following percentages of ambulatory patients: 93% and 85% for mild type I, 29% and 44% for intermediate type IV, and 3% and 9% for severe type III, respectively. However, determination of the exact Sillence type at the time of diagnosis may be occasionally difficult, especially in infants showing sporadic disease manifestations. In addition, recent advances in the molecular findings of OI have complicated the classification of this disease.^[3,8]^ Thus, a simple clinical indicator of disease severity is required for clinical practice. Shapilo proposed another system for classifying the severity of OI,^[9]^ which is based on the timing of the initial fracture. In this system, OI is classified into 4 types: the Congenita A and B types include patients with a fracture at birth, the Tarda A type includes those who did not have a fracture at birth but had a fracture before starting to walk, and the Tarda B type includes those who experienced their initial fracture after starting to walk.^[6,9]^ The age at initial fracture has been reported to be associated with physical function in patient-reported health-related quality of life.^[10]^ According to the Shapilo classification, ambulation ability was low in the Congenita types; 60% of the patients in Tarda A type acquired ambulation; and patients in Tarda B type had the ability to walk. Our results were in agreement with the Shapilo classification since the age at initial fracture in our study was associated with ambulation ability. Thus, this classification system may be a good clinical indicator for predicting future mobility; however, the Tarda A and B types cannot be differentiated early in infancy.

Severe OI often presents with neonatal respiratory failure due to a narrow thorax and multiple rib fractures.^[2]^ We first showed that the presence of neonatal respiratory failure is a negative indicator of future ambulation ability in patients with OI. Respiratory insufficiency and NICU management are directly related to disease severity, although the criteria for NICU management of neonatal respiratory failure vary in each institution. Thus, neonatal respiratory status may be an early indicator of future mobility.

**Figure 1.**
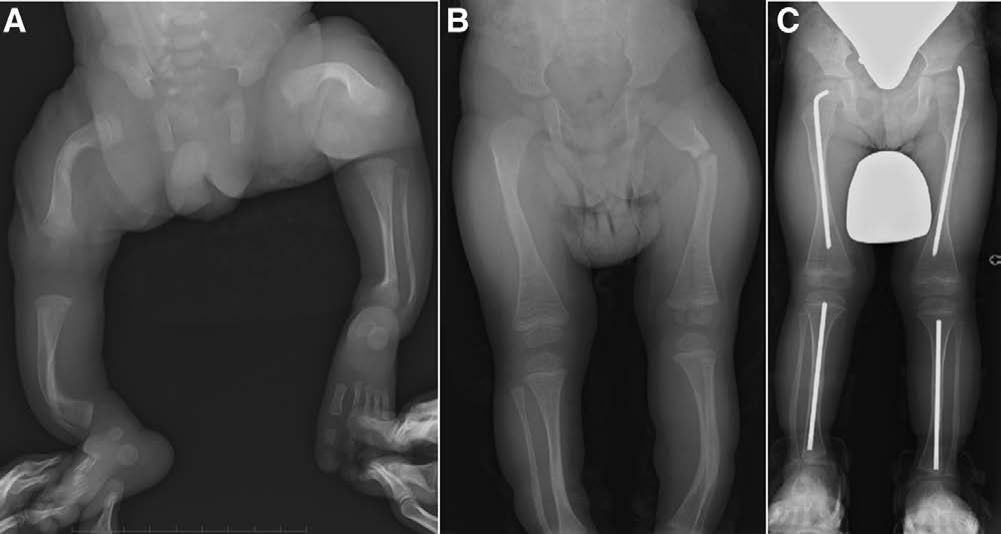
Serial radiographs of an 8-year-old boy with osteogenesis imperfecta. A neonatal anteroposterior radiograph of the lower extremities demonstrating bowing of the long bones of the lower extremities (A) The patient’s birth height was 46.0 cm (−1.3 SD). An anteroposterior radiograph of the lower extremities at the age of 2.1 years showing a left femoral fracture that required corrective osteotomy with intramedullary fixation (B). His height at the first surgery was 78.5 cm (−3.0 SD). An anteroposterior radiograph of the lower extremities at the age of 8.3 years revealing bilateral intramedullary rodding for the femora and tibiae, and the patient was able to walk independently (C).

**Figure 2.**
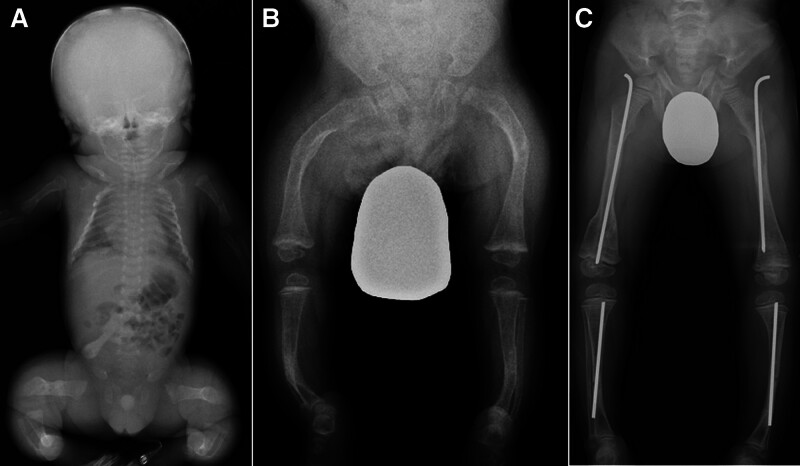
Serial radiographs of a 6-year-old boy with osteogenesis imperfecta. A neonatal babygram demonstrating multiple rib and long-bone fractures of the upper and lower extremities (A) The patient’s birth height was 36.0 cm (−6.2 SD). A preoperative anteroposterior radiograph of the lower extremities at the age of 2.7 years showing bowing of the femora and tibiae with decreased bone density (B). His height immediately before the first surgery was 73.4 cm (−6.6 SD). An anteroposterior radiograph of the lower extremities at the age of 6.0 years showing bilateral intramedullary rodding for the femora and tibiae (C). He has not currently acquired practical walking ability.

In this study, we demonstrated that birth height is associated with future mobility in children with OI. Children with OI are known to be smaller than the normal population, and the height decreases with the severity of the disease.^[11]^ Kruger et al^[12]^ reported that walking distance was correlated with height in patients with severe OI. They also noted that birth height and ambulation ability were also correlated during childhood, indicating that birth height could be a useful indicator for predicting future mobility. In this study, we proposed that a height of −4.0 SD at birth as well as at 3 years of age is the cutoff value for PA ability. We recommend aggressive treatment, such as corrective osteotomy of the lower extremities, to achieve PA in children with a height of −4.0 SD or taller, even before they start walking (Fig. 1). On the other hand, practical walking ability may be difficult to acquire for children with fractures at birth, neonatal respiratory failure, or a birth height of −4.0 SD or smaller (Fig. 2).

The effect of BP medication on patients’ ambulation ability could not be determined in the current study because almost all patients received intermittent intravenous pamidronate administration. Although several reports have demonstrated the effectiveness of corrective osteotomy with intramedullary rodding for lower extremity deformities in patients with severe OI,^[7,13]^ half of the patients who underwent corrective osteotomies were included in the NA group in our study. However, birth height and age at diagnosis were not significantly different between the children who underwent surgery in the PA and NA groups, indicating that disease severity seemed to be similar between the groups. Since the age at surgery was lower in the PA group, some of the children with moderate severity may show future improvement in ambulatory performance if they receive corrective osteotomies in early childhood and subsequently undergo aggressive functional training. We recommend that corrective osteotomy of the lower extremities should be considered around 3 years of age because patients’ future mobility can be predicted to some extent from their height and previous fracture history up to that age.

This study had several limitations. Diagnosis of OI was clinically performed in the majority of patients, and only a few patients underwent genetic testing. The criteria for NICU management and treatment guidelines were not clear, as we have already noted. Due to the retrospective nature of this study, we could not collect sufficient information on physical therapy, which seems to be applicable in most children with OI. In addition, the method of measuring height was not standardized for patients with severe limb deformities who could not keep standing position. Finally, we did not investigate mobility in adolescence and adulthood, which could deteriorate for a variety of reasons, such as decreased activity after graduating from school and maladaptation to the social environment.

## 5. Conclusion

The patient’s height, history of fracture during infancy, and neonatal respiratory status were early predictive factors for future mobility in children with OI. Surgical interventions should be considered around the age of 3 years, and practical walking can be expected to be achieved in children with OI who show moderate disease severity.

## Acknowledgments

The authors would like to thank Editage (www.editage.com) for English language editing.

## Author contributions

Conceptualization: Kenta Sawamura and Hiroshi Kitoh.

Data curation: Kenta Sawamura and Akiko Kitamura.

Formal analysis: Kenta Sawamura and Akiko Kitamura.

Funding acquisition: Hiroshi Kitoh.

Investigation: Kenta Sawamura.

Methodology: Kenta Sawamura and Hiroshi Kitoh.

Project administration: Hiroshi Kitoh and Tadashi Hattori.

Supervision: Hiroshi Kitoh and Tadashi Hattori.

Validation: Kenta Sawamura and Hiroshi Kaneko.

Visualization: Kenta Sawamura and Hiroshi Kaneko.

Writing–original draft: Kenta Sawamura.

Writing–review & editing: Hiroshi Kitoh.

## References

[1] Engelbert RH Pruijs HE Beemer FA. Osteogenesis imperfecta in childhood: treatment strategies. Arch Phys Med Rehabil. 1998;79:1590–4. 9862306 10.1016/s0003-9993(98)90426-9

[2] Forlino A Marini JC. Osteogenesis imperfecta. Lancet. 2016;387:1657–71. 26542481 10.1016/S0140-6736(15)00728-X PMC7384887

[3] Ralston SH Gaston MS. Management of osteogenesis imperfecta. Front Endocrinol. 2020;11:924. 10.3389/fendo.2019.00924 PMC7026366 32117044

[4] Hoffer MM Feiwell E Perry R. Functional ambulation in patients with myelomeningocele. J Bone Joint Surg Am. 1973;55:137–48. 4570891

[5] Sillence DO Senn A Danks DM. Genetic heterogeneity in osteogenesis imperfecta. J Med Genet. 1979;16:101–16. 458828 10.1136/jmg.16.2.101 PMC1012733

[6] Daly K Wisbeach A Sanpera I Jr. The prognosis for walking in osteogenesis imperfecta. J Bone Joint Surg Br. 1996;78:477–80. 8636190

[7] Engelbert RH Uiterwaal CS Gulmans VA. Osteogenesis imperfecta in childhood: prognosis for walking. J Pediatr. 2000;137:397–402. 10969267 10.1067/mpd.2000.107892

[8] Forlino A Cabral WA Barnes AM. New perspectives on osteogenesis imperfecta. Nat Rev Endocrinol. 2011;7:540–57. 21670757 10.1038/nrendo.2011.81 PMC3443407

[9] Shapiro F. Consequences of an osteogenesis imperfecta diagnosis for survival and ambulation. J Pediatr Orthop. 1985;5:456–62. 4019761 10.1097/01241398-198507000-00014

[10] Matsushita M Mishima K Yamashita S. mpact of fracture characteristics and disease-specific complications on health-related quality of life in osteogenesis imperfecta. J Bone Miner Metab. 2020;38:109–16. 31463628 10.1007/s00774-019-01033-9

[11] Jain M Tam A Shapiro JR. Growth characteristics in individuals with osteogenesis imperfecta in North America: results from a multicenter study. Genet Med. 2019;21:275–83. 29970925 10.1038/s41436-018-0045-1 PMC6320321

[12] Kruger KM Caudill A Rodriguez Celin M. Mobility in osteogenesis imperfecta: a multicenter North American study. Genet Med. 2019;21:2311–8. 30918359 10.1038/s41436-019-0491-4 PMC7401984

[13] Rodriguez Celin M Kruger KM Caudill A. A Multicenter study of intramedullary rodding in osteogenesis imperfecta. JBJS Open Access. 2020;5:e20.00031. 10.2106/JBJS.OA.20.00031 PMC7489747 32984750

